# Recognition of skin malignancy by general practitioners: observational study using data from a population-based randomised controlled trial

**DOI:** 10.1038/sj.bjc.6604810

**Published:** 2009-01-06

**Authors:** P Pockney, J Primrose, S George, N Jayatilleke, B Leppard, H Smith, P Little, R Kneebone, A Lowy

**Affiliations:** 1 University Surgery, F Level Centre Block (MP816), Southampton General Hospital, Tremona Road, Southampton SO16 6YD, UK; 2 Southampton Clinical Trials Unit, Mailpoint 131, Southampton General Hospital, Tremona Road, Southampton SO16 6YD, UK; 3 Public Health Sciences and Medical Statistics, Mailpoint 805, Southampton General Hospital, Tremona Road, Southampton SO16 6YD, UK; 4 Department of Dermatology, Southampton University Hospitals NHS Trust, Tremona Road, Southampton SO16 6YD, UK; 5 Elderly Care and Community Medicine, Brighton and Sussex Medical School, Brighton, UK; 6 Primary Medical Care, University of Southampton School of Medicine, Southampton, UK; 7 Department of Biosurgery and Technology, Imperial College, London, UK; 8 Swiss Institute for Applied Cancer Research, 3008 Berne, Switzerland

**Keywords:** skin malignancy, minor surgery, general practice

## Abstract

Skin malignancy is an important cause of mortality in the United Kingdom and is rising in incidence every year. Most skin cancer presents in primary care, and an important determinant of outcome is initial recognition and management of the lesion. Here we present an observational study of interobserver agreement using data from a population-based randomised controlled trial of minor surgery. Trial participants comprised patients presenting in primary care and needing minor surgery in whom recruiting doctors felt to be able to offer treatment themselves or to be able to refer to a colleague in primary care. They are thus relatively unselected. The skin procedures undertaken in the randomised controlled trial generated 491 lesions with a traceable histology report: 36 lesions (7%) from 33 individuals were malignant or pre-malignant. Chance-corrected agreement (*κ*) between general practitioner (GP) diagnosis of malignancy and histology was 0.45 (0.36–0.54) for lesions and 0.41 (0.32–0.51) for individuals affected with malignancy. Sensitivity of GPs for the detection of malignant lesions was 66.7% (95% confidence interval (CI), 50.3–79.8) for lesions and 63.6% (95% CI, 46.7–77.8) for individuals affected with malignancy. The safety of patients is of paramount importance and it is unsafe to leave the diagnosis and treatment of potential skin malignancy in the hands of doctors who have limited training and experience. However, the capacity to undertake all of the minor surgical demand works demanded in hospitals does not exist. If the capacity to undertake it is present in primary care, then the increased costs associated with enhanced training for general medical practitioners (GPs) must be borne.

The United Kingdom has a health service divided into two distinct components. The large majority of people are registered with a GP who is the first point of contact with the service. There is no direct route to secondary care except through private consultation for a minority, or through the emergency room. Ever since the 1990 contract for GPs in England and Wales that specified an item of service payment for minor surgical procedures, there has been fierce debate about the quality and appropriateness of management decisions and clinical practices in general practice, focussing around two issues (Paraskevopoulos *et al*, 1988; Department of Health & The Welsh Office, 1989; Brazier and Lowy, 1991; Bull *et al*, 1991; McWilliam *et al*, 1991; Brown *et al*, 1992; Cox *et al*, 1992; Bricknell, 1993; Lowy *et al*, 1997, 1998; Cross, 1998; Khorshid *et al*, 1998; Kirby *et al*, 1998; Suvarna *et al*, 1998). First is the technical quality of surgery performed, discussed most often in terms of incomplete excision of malignant or pre-malignant conditions, and which we have addressed in a population-based randomised controlled trial of minor surgery (George *et al*). Intimately associated with this is the second issue, the accuracy of clinical diagnosis and the consequent need for histological confirmation of diagnosis. Until now, there has been a relative absence of firm UK evidence: what evidence exists comprises descriptions of personal case series from general practice, and audits of completely or incompletely excised lesions reported by pathologists, with or without accurate diagnoses recorded on the pathologist's request form.

In a population-based randomised controlled trial of minor surgery, we identified that GPs make less use of pathology services than do hospital doctors (George *et al*). We have been unable to identify population-based studies comparing clinical diagnosis in primary care and laboratory histology in the United Kingdom. This paper aims to establish how well GPs recognise skin cancer, using data from our trial.

## Materials and methods

### Data

This study used data on pathological samples collected during a population-based randomised controlled trial comparing the quality of minor surgery performed by GPs and hospital doctors (George *et al*). All patients recruited to the trial had a GP referral form indicating a working diagnosis for the lesion concerned. These working diagnoses were entered into a database, along with the histological diagnosis found on the histology form pertaining to the sample, where one was found. Multiple searches were undertaken in each of the pathology departments serving the area from which patients were recruited over the whole period of the trial to find these reports. There were many diagnoses on both referral forms and pathology reports, and we divided them into 23 categories, using a classification derived from Rook's Textbook of Dermatology, Sixth edition (Champion *et al*, 1998). The categories arrived at are shown in Tables 1 and 2. We excluded ingrowing toenails from all aspects of the ensuing analysis, leaving 22 categories of lesion. These were further collapsed into ‘benign’ and ‘malignant’ categories for analysis. The ‘malignant’ category comprised three malignancies (malignant melanoma, squamous cell carcinoma and basal cell carcinoma), one low-grade malignancy (keratoacanthoma) and one pre-malignant condition (Bowen's disease).

### Analysis

Chance-corrected, inter-rater reliability was measured using Cohen's *κ* in Stata 9.0 (Stata Corp). A *κ* value greater than 0.75 is considered excellent agreement beyond chance, values below 0.40 represent poor agreement, and values between 0.40 and 0.75 represent fair-to-good agreement (Fleiss, 1981). The sensitivity, specificity and positive predictive value for GPs’ recognition of skin malignancies were calculated with 95% confidence intervals (CIs). We did not run a sensitivity analysis on the data as missing cases greatly outnumbered known malignancies, and it was felt that the assumption that all missing cases were malignant was unlikely. In the group of malignancies in which surgery was undertaken by the GP, we used cross-tabulation to examine whether recognition of the lesion as malignant had an effect on completeness of excision.

## Results

Five hundred and sixty-eight individuals entered the trial, which generated these data by 82 GPs. Their average age was 48.75 years, and 309 of them (54.4%) were women. Sixty-five GPs undertook surgery in the primary care arm of the trial and 60 hospital surgeons or dermatologists in the hospital arm. Of 705 lesions, 654 can be shown to have been subject to a procedure, 17 of these involving ingrowing toenails in which histology is not usually performed and which were excluded from later analysis. Overall, 491 of the 637 skin procedures (77%) generated a traceable pathology report. Table 1 shows numbers of these cases by diagnosis as described by the GPs and number in each category in which a histological sample was found by trial arm. In one case, there was no referral from the GP, the procedure having been performed at the request of a patient with multiple lesions and in whom that lesion had not been mentioned in the referral. This lesion was excluded, leaving 490 for further analysis. The table demonstrates that the deficit in samples does not follow a random pattern. However, although it might be expected that skin tags would be under-represented, shortfalls in other categories, which can sometimes closely resemble malignancies (e.g. basal cell papillomata, melanocytic naevi), are more worthy of concern. Table 2 shows the numbers of cases in which a histological sample was found as described by GPs and as classified by histological examination (ingrowing toenails excluded).

### Agreement between GP diagnosis and histology

An overall *κ* statistic of 0.45 (95% CI, 0.36–0.54) was obtained for agreement between GP diagnosis and later histology. Even at its upper 95% CI, therefore, agreement is moderate at best. Four of the lesions (all basal cell carcinomas) were diagnosed, correctly, in the same individual, and it may be possible that the finding of one malignancy lesion pre-disposes the examiner to find others. If *κ* is recalculated to reflect individuals correctly diagnosed with malignancy, rather than lesions, the resulting statistic is 0.41 (0.32–0.51). Again, even at the upper level of statistical confidence, agreement is ‘moderate’ at best.

### Test characteristics of GPs in detecting skin malignancy

The results above can be expressed as 2 × 2 tables and test characteristics (sensitivity, specificity, predictive values) were computed. Sensitivity is calculated as the proportion (or percentage) of malignancies correctly diagnosed, whereas specificity is the proportion (or percentage) of non-malignancies correctly diagnosed. A positive predictive value is the proportion of positive diagnoses that correctly identify a malignancy and a negative predictive value the proportion (or percentage) of negative diagnoses that correctly exclude one. Table 3 shows the data for individual lesions, with test characteristics computed below, and Table 4 is the analogous table for individuals affected with malignancy. The results do not differ a great deal between the two analyses. They indicate that, in our population, GPs failed to recognise one-third of the skin malignancies, or slightly more than one-third of the patients with malignancies. Taking statistical uncertainty into account, the upper 95% CI for both analyses indicates that they miss no fewer than one in five. Neither of the malignant melanomas included here was diagnosed by the GP concerned: one was described as a ‘dermatofibroma’ and the other given a general description as ‘red lesion’. A further two malignancies do not form part of this data set: although randomised they were lost to follow-up because hospital doctors judged that they did not meet an inclusion criterion, which was that the GPs should feel to be able to offer treatment themselves or to be able to refer to a colleague in primary care. Neither case was felt to be suitable for treatment within the context of a hospital minor surgery unit, and both were referred for specialist treatment. One was a squamous cell carcinoma in a ‘difficult’ area and the other a large malignant melanoma that had presented on multiple occasions before referral.

## Discussion

Not all malignant lesions are clinically obvious at presentation, and some have potentially serious adverse outcomes if missed. In this study, GPs missed a third of malignancies, including both malignant melanomas, and two further malignancies were excluded at an earlier stage because of the lack of recognition of what they were. Clearly, more study is required: these data were collected in the highly artificial environment of a controlled trial and, despite some evidence to the contrary, it may be that all the malignancies unrecognised here would have been referred to specialist care under a 2-week waiting rule in a real-world situation. However, although confined to one geographical area of the United Kingdom, this study was population based and included patients referred by a large number of GPs, and we believe the results to be generalisable. It is reassuring for us, but perhaps not for patients, that our results are echoed in studies from other countries. Whited *et al* (1997) showed that primary care clinicians identified the presence of skin cancer with a sensitivity of 57% (95% CI, 44–68%) in one US study and concluded that ‘Without improved diagnostic skills, primary care clinicians’ examinations may be ineffective as a screening test’. Youl *et al* (2007) in Australia found that although, overall, sensitivity for diagnosing any skin cancer was similar for skin cancer clinic doctors (94%) and GPs (91%), sensitivity was higher for skin cancer clinic doctors for BCC (89 *vs* 79%; *P*<0.01) and melanoma (60 *vs* 29%; *P*<0.01). Raasch (1999), again in Australia, showed a sensitivity of 69.1% (95% CI, 62.5–75.7%) for primary care doctors in a series of non-melanoma skin malignancies. But what should the sensitivity be? Perfection, after all, is not easy to attain. True comparisons between dermatologists’ and primary care physicians’ accuracy in diagnosing melanoma are uncommon, but one systematic review showed a bottom end of the range of values of sensitivity to malignant melanoma in dermatologists of 81%, but of only 41% in primary care physicians (Chen *et al*, 2001). In the face of rising skin cancer incidence it is clear that the major challenge of providing minor surgery in primary care is the potential for missed diagnosis of serious skin malignancies (Diffey, 2004).

The 1990 contract was changed in 2004 so that there are stringent standards in place for those wishing to become general practitioners with a special interest in dermatology (Shekelle, 2003). However, these individuals, analogous to the ‘skin cancer clinic doctors’ in the study by Youl *et al* (2007), are not sufficient in number to undertake the assessment of all minor skin lesions presenting in primary care, and hospital services do not have the capacity either. GPs who merely wish to be added to the minor surgery list now have to be signed off by their trainer as competent, competent, but standards are set locally. Clinical guidelines issued by NICE make recommendations for referral of patients with suspected cancer from primary care to specialist services (National Institute for Health and Clinical Excellence, 2006). These guidelines recommend that patients presenting with skin lesions suggestive of skin cancer, or in whom a biopsy has been confirmed, should be referred to a team specialising in skin cancer. However, it is not clear what will happen if GPs do not suspect that a lesion is cancerous, as in one-third of the malignancies in this study.

These results place an important ‘health warning’ around the assumption that shifting services from secondary care to primary care carries only benefits (Department of Health, 2006). There is not the capacity in hospitals to take on the workload of minor surgery or even of mere diagnosis of all skin lesions, and it would likely be unpopular with patients if it were to happen (George *et al*). We do not believe that the background of a doctor in general practice, surgery or even dermatology is fundamentally important, but we do believe that it is unsafe to leave the diagnosis and treatment of potential skin cancer entirely in the hands of doctors who have had insufficient training to do it. The increased costs associated with developing and delivering appropriate training to all GPs, not just for those with a specialist interest, must be acknowledged and provided.

## Figures and Tables

**Table 1:** Number of cases as described by GPs on referral form, numbers in whom a procedure can be shown to have been performed and numbers of those in whom a histological sample was found, by trial arm

			**Histological sample found**	
**GP's description**	**Total with GP diagnosis**	**Total operated upon**	**Hospital group**	**Primary care group**
*Lesions analysed*				
1 Unknown/nonspecific description	14	14	6/7	7/7
2 Eczema/dermatitis	0	0	0/0	0/0
3 Granuloma	8	6	4/4	2/2
4 Solar elastosis	0	0	0/0	0/0
6 Sebaceous gland hyperplasia	1	1	0/0	1/1
7 Skin tag, fibroepithelial polyp, skin polyp	58	55	14/20	10/35
8 Chondrodermatitis nodularis helices	0	0	0/0	0/0
9 Viral warts	12	12	5/5	7/7
10 Scars including keloid	2	2	1/1	1/1
11 Benign tumours including neurofibroma	32	30	13/13	12/17
12 Lipoma	19	17	6/7	4/10
13 Trichilemmal cysts and epidermoids	157	143	74/90	30/53
14 Lentigo	0	0	0/0	0/0
15 Seborrhoeic keratosis, seborrhoeic wart, BCP	148	138	57/79	43/59
16 Melanocytic naevus	159	150	66/72	62/78
17 Solar keratosis	4	4	2/2	2/2
18 Cutaneous horn	1	1	0/0	1/1
19 Bowen's disease	1	1	1/1	0/0
20 Basal cell carcinoma	51	45	21/21	23/24
21 Keratoacanthoma	4	4	3/3	1/1
22 Squamous cell carcinoma	8	7	3/3	4/4
23 Malignant melanoma	4	4	1/1	3/3
Total	683	634	277/329 (84%)	213/305 (70%)
				
*Lesions not analysed*				
5 Ingrown toenail	18	17	0/9	0/8
No procedure undertaken or referred elsewhere			28	23
Not referred by GP	2	1	1/1	0/0
No data on referral form	2	2	0/2	0/0
**Grand Total**	705	654	369	336

GP=general practitioner.

**Table 2:** Comparison of numbers (%) in each histological category as described by GPs and as classified by histological examination (ingrowing toenails not displayed)

	**GP diagnosis**	**Histological diagnosis**
1 Unknown/nonsense description	13 (2.6)	0
2 Eczema/dermatitis	0	2 (0.4)
3 Granuloma	6 (1.2)	12 (2.4)
4 Solar elastosis	0	9 (1.8)
6 Sebaceous gland hyperplasia	1 (0.2)	1 (0.2)
7 Skin tag, fibroepithelial polyp, skin polyp	24 (4.7)	27 (5.3)
8 Chondrodermatitis nodularis helices	0	1 (0.2)
9 Viral warts	12 (2.4)	15 (2.9)
10 Scars including keloid	2 (0.4)	1 (0.2)
11 Benign tumours including neurofibroma	25 (4.9)	64 (12.6)
12 Lipoma	10 (2.0)	11 (2.2)
13 Cysts including epidermoids	104 (20.4)	72 (14.1)
14 Lentigo	0	7 (1.4)
15 Seborrhoeic keratosis, seborrhoeic wart, BCP	100 (19.6)	93 (18.5)
16 Melanocytic naevus	128 (25.1)	134 (26.3)
17 Solar keratosis	4 (0.8)	4 (0.8)
18 Cutaneous horn	1 (0.2)	0
19 Bowen's disease	1 (0.2)	3 (0.6)
20 Basal cell carcinoma	44 (8.6)	26 (5.1)
21 Keratoacanthoma	4 (0.8)	1 (0.2)
22 Squamous cell carcinoma	7 (1.4)	5 (1.0)
23 Malignant melanoma	4 (0.8)	2 (0.4)
**Total**	490	490

GP=general practitioner.

Figures in the second column are not a subset of figures in the first column.

**Table 3:** Benign and malignant skin lesions as judged by histology and by GP diagnosis

	**Histology malignant**	**Histology benign**	**Total**
GP diagnosis malignant	24	36	60
GP diagnosis benign	12	416	428
Total	36	452	488

GP=general practitioner.

Sensitivity=66.7% (52.9–78.00).

Specificity=92.0% (89.7–93.9).

Positive predictive value=40% (30.2–50.6).

Negative predictive value=97.2% (93.6–98.2).

**Table 4:** Individuals classified by whether they were judged to have a malignant skin lesion as judged by histology and by GP diagnosis

	**Histology malignant**	**Histology benign**	**Total**
Malignant diagnosis by GP	21	35	56
No malignant diagnosis by GP	12	355	367
Total	33	390	423

GP=general practitioner.

Sensitivity=63.6% (49.3–75.4).

Specificity=91.0% (88.5–93.1).

Positive predictive value=37.5% (27.6–48.5).

Negative predictive value=96.7% (94.8–98.0).
